# Supplementary material for: Achievements and challenges of lymphatic filariasis elimination in Sierra Leone
Source: PLoS Negl Trop Dis. 2020 Dec 28;14(12):e0008877. doi: 10.1371/journal.pntd.0008877 (PMC7793261; doi:10.1371/journal.pntd.0008877)
Supplement: Attachment — Submitted filename: Comments and responses R1.pdf [file pntd.0008877.s003.pdf]

Achievements and challenges of lymphatic filariasis elimination in Sierra Leone

Authors responses to comments

Editor's remarks:
One reviewer has in particular raised concerns regarding the novelty and suitability of the work as a research article for publication in PLoS NTDs.
Because there are many precedents of similar epidemiological surveys pre and post elimination programmes in the PLoS NTDs archive, I do not necessarily agree that this epidemiological survey is unsuitable for publication in PLoS NTDs.
Response: Thank you. We very much appreciate your comment on the suitability of publishing such program data in this journal. These are very important experience in countries that face different challenges to eliminate LF. By sharing such information, countries could learn from each other to achieve the global goal of LF elimination.

However, the reviewer also queries whether the data set is novel. As it comprises outputs of the LF programme in Sierra Leone including MDA coverage, TAS survey results, and pre-TAS results, the data may have already been put into the public domain elsewhere. The pilot data surveying *Mansonella* co-prevalence, is not, in my judgement, sufficiently robust for standalone consideration.
I therefore query with the authors whether the data is unique or has already been published elsewhere? Please consider this carefully in any response to review.
Response: Thank you. Except the baseline prevalence data we cited from Ref #9, all data presented in this paper have not been published or submitted elsewhere for peer-reviewed publication.

Could additional analyses be provided to provide further novelty, on clustering effect of TAS positive cases, for instance, as suggested by reviewer 1?
Response: Thank you. We added analysis on the clustering effect of the TAS positive cases as suggested.

Please also carefully attend to reviewers' critiques on data presentation, especially reviewer 3.
Response: Thanks. Please see responses to the relevant comments.

Reviewer's Comments:

<u>Reviewer #1:</u>
The objectives of the study are not clearly articulated in the methods section, a clear testable hypothesis is not stated. The methods section details the steps the country has taken towards LF elimination as per the GPELF requirements: MDA, coverage assessments, pre-TAS, TAS. The methods section does not provide details of any further statistical analysis, beyond routine reporting of programmatic data.
Response: The objectives are described in the last paragraph of the Introduction. This paragraph is now revised to reflect the hypothesis that after the number of the rounds of MDA the districts should have reached the respective objectives. The surveys were to test whether this was the case. The coverage was evaluated effective or ineffective against the WHO recommended thresholds of 65% for epi coverage and 80% for program coverage. Comparison between different calculations was not the purpose of the analysis, therefore not necessary. The Methods section has also been revised.

The MDA epidemiological and programmatic coverage is calculated using three different population sources, it is not clear why these three population sources were selected. Other than presenting the MDA coverage using these three different population sources, there is no analysis presented in the methods to

look at differences between the population sources, and the impact this has on achieving the necessary programmatic MDA coverage.

Response: The accuracy of denominators is a longstanding issue in endemic countries. The purpose of using different sets of denominators was to evaluate the treatment coverage as effective or ineffective against the WHO recommended minimum thresholds, not to compare the different denominators. Please see the response above.

In the ethical approval section of the methods, it states that 'participants identities were protected by collecting, recording and analyzing data such that participants remained anonymous'. However, when detailing where the data can be found, the authors state that 'data is available with certain restrictions due to the patients personal information contained in the data.'

Response: Thanks for pointing this out. The language in the Ethics section is revised.

The results section is very limited, with just over a page of results presented. The results presented are not clear or completely presented. For example, when presenting the MDA coverage results by district - the author refers to districts reporting ineffective coverage 'on a total of 5 of 6 occasions'.

Response: The results are shown in text, figures and tables. We do think that the results are sufficient for this paper. However, we have edited the text and data presentation in conjunction with comments from other reviewers. We believe this is now better presented.

From table 1, it is evident that districts with a high baseline prevalence (Kono and Tonkolili), have the greatest number of FTS positive cases identified during TAS. However, no analysis is undertaken to determine the impact of MDA on the LF prevalence - the results only present the MDA data (using three population sources), and the TAS/pre-TAS results.

Response: We appreciate this comment. Table 1 shows the TAS results in 8 districts. The results are judged on whether the Evaluation Units passed the TAS according to the critical cutoff values (WHO Guidelines). As the survey methodologies were different in conducting TAS and the baseline survey, the results are not directly comparable, therefore no analysis on comparison is conducted here. However, the 8 districts successfully passed the pre-TAS which qualified these districts for conducting TAS. Such pre-TAS results were comparable with baseline and the comparison was made and published already in ref #15 quoted in the paper.

From the map presented in Figure 2 - it is not clear what the cluster represents - is it the location of the household of the positive case or the school? It would be interesting to understand the clustering effect of TAS positive cases - from how many schools did the 7 FTS positive cases identified in Kono and Tonkolili come from? Was information collected during the TAS to determine if these positive individual took the MDA? If so, what were the reasons for not taking MDA? How does the LF programme intend to use these results to plan further surveillance activities.

Response: The TAS survey here was a school-based cluster survey as described in the Methods. Each cluster represents a school surveyed. We have conducted the clustering analysis using the Spatial Autocorrelation (Moran's Index) in ArcGIS. This is added to the results.

The title of the paper is 'achievements and challenges of LF elimination in Sierra Leone' yet no reference is given to Sierra Leone's achievements and challenges towards the second pillar of the GPELF: morbidity management and disability prevention for lymphodema and hydrocele patients.

Response: We appreciate this valuable comment. We have added this in the Discussion as a critical challenge for Sierra Leone.

In the discussion, the authors present their 'program observation' to justify potential reasons why the five districts failed pre-TAS, and four districts failed the pre-TAS twice. This is anecdotal evidence and it is not clear how these conclusions have been reached.

Response: These are discussions on potential reasons, not conclusion on the reasons of pre-TAS failure. The exact reasons are hard to know in a national program. Only when you consider all potential reasons and start to address these in programming, can the program evolve and achieve the program goals. There is only one "program observation" cited in the coverage discussion. We believe such discussions are justified.

This paper presents the output of the LF programme in Sierra Leone including MDA coverage, TAS survey results, and pre-TAS results. This data presented is not novel and the authors do not conduct any further analysis to explore reasons for pre-TAS failure, other than anecdotal evidence presented in the discussion.
Response: We do not agree with the Reviewer on this comment. The paper presents the outcome of a national LF elimination program and discuss the challenges to achieve the program goal. Only through transparent dissemination and discussion of the program achievements and challenges the national programs are facing, can we learn from each other and overcome the challenges to achieve the global goal of elimination of LF. Every country experience is valuable in the cause. This is not a pure research.

Reviewer #2:
This manuscript is well written and reports important outcomes that are highly relevant to the NTD community. The study design and methodology are appropriate for the research question. However, the narrative could be better structured to enhance its impact.
Response: Thanks for this comment.

Major comment
The title does not project the value of progressive outcomes that will transition to elimination of LF in Sierra Leone. This manuscript could be the first of three papers in a series titled 'Towards Elimination of LF in Sierra Leone as suggested below:
I. Towards elimination of LF in Sierra Leone 1: Cross border challenges
II. Towards elimination of LF in Sierra Leone 2: MDA to eliminate LF during public health emergencies
III. Towards elimination of EL in Sierra Leone 3: Interruption of LF transmission nationwide
The 'conclusions/significance' section in the abstract ties in well with the suggested title for the first series: 'Eight districts in Sierra Leone have successfully passed TAS1 and stopped MDA, with one more district qualified for conducting TAS1, a significant progress towards LF elimination. However, great challenges exist in eliminating LF from the whole country with repeated failure of pre-TAS in border districts. Effort needs to be intensified to achieve LF elimination.
The references to carrying out MDA during the Ebola epidemic should be expanded into a long-awaited story of the contribution of the CDDs in the containment of Ebola in Sierra Leone. This should be a separate paper that explores the following questions: Why were TAS surveys stopped during the Ebola epidemic? How did CDDs contribute to contact tracing and MDA for malaria? What led to the reduced CDD motivation after the Ebola epidemic? It is clear that the data and information to address these questions are available.
Response: Thanks for this suggestion. We did think about going this way before we drafted the paper, but looking at the data we have, we decided to combine into one paper as separately the data would not have justified either papers for PLoS NTDs (Reviewer #1 already criticized the limited data in this paper). But certainly, the third paper will be considered when Sierra Leone achieved interruption of LF nationwide.

Minor comments
Abstract
Line 3: replace 'every' with 'all 12 districts' to and do the same in the main background. It is a bit confusing, if this clarification is not made from the outset.
Response: Done.

Introduction
Paragraph 1, Line 3: replace ‘due to’ with ‘manifested as’.
Paragraph 2, line 1. Why start with 2014? I would limit this to figures for 2017.
Response: Done and revised.

Ethical approval
Crosscheck that approval was obtained from MOHS Research and Ethics Committee and not the National Ethics Committee.
Response: Thanks. Correct.

Reviewer #3:
The topic of the paper is very interesting and important since countries are facing challenges with transmission assessment surveys in some of the settings
Response: Thanks for the comment.

Following comments are from the attached document.

Transmission assessment surveys for lymphatic filariasis are recommended by the World Health Organization (WHO) as a mean to evaluate the impact of mass drug administration on the transmission of the infection. This topic is important since countries endemic for lymphatic filariasis do fail these assessments in some settings for a few reasons. I liked reading your paper and I am happy to share few comments
Response: Thanks for the comment and glad you liked this paper.

INTRODUCTION.
Th introduction section is straight to the point and ends well with a clear aim of the paper which is to present the results of surveys conducted in the context of their mass drug administration and discuss related challenges. However, I do have some minor comments on few sentences in this section.

1. The first comment is on the use of abbreviation or acronyms. I suggest writing in full the word followed by the abbreviations and acronyms in bracket for the first time and next time just use the acronyms and abbreviations. For example, in the following sentence, “WHO recommends that 5-6 rounds of effective annual MDA be conducted to eliminate LF transmission., I *suggest the following “The World Health Organization (WHO) recommends be conducted to interrupt the transmission of LF “*.

Response: Thank you. This has been checked and addressed throughout the paper.

2. You mentioned in the following sentence the ineffectiveness of CDTI: “In 2010 a distribution strategy based upon immunization campaign was introduced in the remaining districts Western area urban and rural due to the ***ineffectiveness*** of the CDTI.”
   First, I am wondering what you mean by infectiveness of CDTI. I guess you are referring to challenges related to the implementation of CDTI in urban areas compared to rural areas. Secondly, I think this statement does not match with the reference provided which refers to high coverage of MDA of lymphatic filariasis which is in contradiction with the effectiveness of CDTI. Could you please look at this?

Response: Your assumption was correct. It is the approach using CDDs was not effective. We have revised the description as follows: “In 2009, the CDTI plus albendazole approach using CDDs in the remaining 2 districts Western Area Urban and Rural was found to be ineffectual in these urban/rapidly urbanizing settings attaining only 29% epidemiological coverage. So, in 2010 a distribution strategy based upon a five day-immunization campaign was introduced implemented by paid community health workers/trainee health workers [12].”

Methods

3. On this section, I think there is a need having a brief introduction for this section. Could you please present in one or two sentences the methods used in your paper?

Response: As the details are given in each method sections, it is unnecessary to have introduction sentences at the beginning of the Section. We decided not to add.

4. I think the first two headings of your methods section "**mass drug administration**" and "**epidemiological and programmatic coverage assessments**" could be brought back to the introduction. In the revised introduction section, you can briefly mention what was done in terms of Mass drug administration and treatment coverage. Since you clearly mentioned that epidemiological and programmatic surveys were published previously, this support also the suggestion of mentioning what was done in terms of epidemiological and programmatic coverage assessments in the introduction section.

Response: Agreed. Most of these two sections have been moved and modified to fit into the Introduction. However, as the coverage data were presented in the Results, we therefore added a subheading as "MDA coverage data collection" to describe how these data were collected. We moved the calculation of coverage to the Data analysis section.

5. You used in these section, the following acronyms/abbreviations **CHW**s"; "**re-pre-TAS**", "NTDP ", "MOHS ", "**s**" . "**hr**"? What does each of them stand for? Please kindly refer to my comment on the use of acronyms and abbreviations (comment #1).

Response: Please refer to the response to Comment #1 above. The abbreviations are now clearly defined. "s", "min" and "h" are standard abbreviations for time, but these are now made in full.

RESULTS

TAS in eight districts

6. Could you please add the year of the survey in the title of table #1?

Response: this is modified to "Results of transmission assessment surveys in eight districts in four EUs in Sierra Leone in 2017"

7. You mentioned that the number of FTS positive was well below the critical cut-off value for each evaluation unit as shown in Figure #2. I suggest that you add Table 1 as well

Response: thanks. This is corrected and Table 1 is mentioned in the previous sentence.

8. You mentioned that 49.3% and 50.7% of children examined during TAS were males and females respectively. No reader can see that in your table. Could you please split the column on "number of persons tested per EU" in three sub columns, one for males, one for females and the third one on total. This shows to show approximately the number of males and females to readers the above-mentioned statistics with regards to gender? (see suggestion below)

Response: Table 1 is amended by sex as requested.

Re-pre-TAS in four districts and pre-TAS in two districts

9. The total of 3,994 persons is for districts targeted for Pre-TAS (4) and Re-pre-TAS (2) not for four districts during re-pre-TAS as highlighted in the paper. I suggest that you split the number in two and provide the number of people targeted for Re-Pre-TAS in four EUs and number of people targeted for Pre-TAS in two EUs.

Response: Thank you, amended as suggested and the total sample 3,994, comprising 2,744 for the repeat pre-TAS and 1,250 for the pre-TAS is now clarified in the text.

10. Could you please refer to my comment# 8 to review table #2?
    Response: *Table 2 amended to show sample and results by sex.*

11. You presented the results of Pre-TAS and referred to table #3. Where is table #3? Were you referring to figure #3? If yes please kindly correct table #3 to figure #3.
    Response: Corrected to table 2.

12. Regarding Figure #3, one spot check village with FTS prevalence of 2.9 is missing in the figure. Could you please kindly add it?
    Response: This was due to an error in coordinates and is now corrected. We also added two other missing site in the Western Area Urban.

13. With regards to pre-TAS results, you mentioned the individual antigenemia prevalence was 0.7% and 0.7% in West Area Urban qualifying for conducting TAS and 1.7% and 3.7% in Western Area rural failing to qualify for TAS as shown in **Figure #3**. I suggest you mention the table number as well after splitting your table #2 in two as I suggested above.
    Response: Corrected and Table 2 (split by sex) is now mentioned

14. To differentiate Pre-TAS and re-pre-TAS in figure #3, could you please different colors?
    Response: *different background colors for repeat pre-TAS and TAS now used*

Microfilaria detection in the FTS positive cases

15. The number of positive FTS in the four districts targeted for Re-pre-TAS is 278 not 296. Could you please check and confirm the provided number (296) in the paper?
    Response: The total number FTS positive in the repeat failed pre-TAS districts was 277. This was an oversight and is now corrected.

DISCUSSION

16. You mentioned that only four districts failed the Pre-TAS. How about Western area rural?
    Response: Corrected, four districts failed the repeat pre-TAS

17. You mentioned in your discussion section, key factors which could explain the ongoing transmission in Kailahum, Kenema, Koinadugu, Bombali and Western rural areas, which are high baseline prevalence in the border districts, traditional beliefs, treatment coverage issues due to population movement within the country/across borders, inaccurate denominators, internal migration, movement of pastoralists from the fullah ethnic groups, difficulty to keep to the appropriate treatment time, demotivation of CDDs, fear of side effects by communities. Do you have an idea of vectors species involved in the transmission of the infection? The knowledge of vectors responsible of the transmission is extremely important since not all of them have the same competence. Which species do you have in settings that passed TAS and which ones do you have in those that failed Pre-TAS and Re-pre- TAS?

Response: We have made new references to vector competence in the Discussion and Conclusions.

'Higher baseline prevalence is possibly related to higher vector competence and redisposing socio-economic circumstances or occupations of the population [23]. The principal mosquito vectors of LF in West Africa of the Anopheles gambiae complex with Culex species playing only a minor if any role. Culex has been found to be the predominant species in the Western Area Urban setting and the South: Bo and Pujehun districts [24]. The importance of vector competence and biting rates contributing to LF hotspots despite repeated effective rounds of MDA has been described in Ghana [25]. The challenges experienced in Sierra Leone are similar to other countries approaching LF elimination targets [26].'

Supported by 4 references:

23 de Souza DK, Koudou BG, Bolay FK, et al. Filling the gap 115 years after Ronald Ross: the distribution of the Anopheles coluzzii and Anopheles gambiae s.s from Freetown and Monrovia, West Africa. PLoS One. 2013;8(5):e64939. Published 2013 May 31. doi:10.1371/journal.pone.0064939
24. de Souza DK, Sesay S, Moore MG‐et al. No Evidence for Lymphatic Filariasis Transmission in Big Cities Affected by Conflict Related Rural-Urban Migration in Sierra Leone and Liberia. PLoS Negl Trop Dis. 2014 Feb 6;8(2):e2700. doi: 10.1371/journal.pntd.0002700. eCollection 2014 Feb.
25. Pi-Bansa S, Osei JHN, Frempong KK, et al. Potential factors influencing lymphatic filariasis transmission in "hotspot" and "control" areas in Ghana: the importance of vectors. Infect Dis Poverty. 2019;8(1):9. Published 2019 Feb 5. doi:10.1186/s40249-019-0520-1
26. Burgert-Brucker CR, Zoerhoff KL, Headland M, et al. Risk factors associated with failing pre-transmission assessment surveys (pre-TAS) in lymphatic filariasis elimination programs: Results of a multi-country analysis. PLoS Negl Trop Dis. 2020;14(6):e0008301. Published 2020 Jun 1.

18. The World Health Organization recommends integrated vectors control measures in the fight against lymphatic filariasis as alternative measures to combat Lymphatic filariasis. These include IRS and use of long-lasting insecticide treated bed nets (LLITNs). Do you have an idea of the use of IRS and LLITNs in these districts which passed TAS and in those where the transmission is still ongoing?

Response: Discussion on IRS and LLITNs has been included.
'An evidence-led vector control program is led by the National Malaria Control Program: four regional hubs provide annual information on vector composition, behavior, susceptibility to insecticides and long-lasting insecticide treated nets (LLITN) longevity and effectiveness. However, despite several rounds of universal distribution the use of LLITN remains low in Sierra Leone. In 2018, the percentage of households with at least one LLITN for every two persons was higher in rural than urban areas (28% versus 21%) and higher in the Eastern and Southern provinces than in other provinces (range 18%-29%) [32]. Public-Private partnership to scale up the use of Insecticide Residual Spraying (IRS) as recommended by WHO has not been implementation at scale after a trail period in Bo district in 2012 [33].'

Supported by 2 references:

Statistics Sierra Leone, Demographic and Health Survey. 2019, p35.
Ministry of Health and Sanitation, National Malaria Control, 2019, p48.
www.afro.who.int/publications/sierra-leone-malaria-control-strategic-plan-2016-2020

CONCLUSION

19. What are the limitations of your study?

Response: Study limitations have been added.
