# Supplementary material for: Achievements and challenges of lymphatic filariasis elimination in Sierra Leone
Source: PLoS Negl Trop Dis. 2020 Dec 28;14(12):e0008877. doi: 10.1371/journal.pntd.0008877 (PMC7793261; doi:10.1371/journal.pntd.0008877)
Supplement: Attachment — Submitted filename: Achiements and challenges of LF in SL.docx [file pntd.0008877.s002.docx]

**Review of the Paper: Achievements and challenges of lymphatic filariasis elimination in sierra Leone**

Transmission assessment surveys for lymphatic filariasis are recommended by the World Health Organization (WHO) as a mean to evaluate the impact of mass drug administration on the transmission of the infection. This topic is important since countries endemic for lymphatic filariasis do fail these assessments in some settings for a few reasons.

I liked reading your paper and I am happy to share few comments

**INTRODUCTION**.

Th introduction section is straight to the point and ends well with a clear aim of the paper which is to present the results of surveys conducted in the context of their mass drug administration and discuss related challenges. However, I do have some minor comments on few sentences in this section.

1. The first comment is on the use of abbreviation or acronyms. I suggest writing in full the word followed by the abbreviations and acronyms in bracket for the first time and next time just use the acronyms and abbreviations. For example, in the following sentence, “WHO recommends that 5-6 rounds of effective annual MDA be conducted to eliminate LF transmission., I *suggest the following “The World Health Organization (WHO) recommends be conducted to interrupt the transmission of LF “.*
2. You mentioned in the following sentence the ineffectiveness of CDTI: “In 2010 a distribution strategy based upon immunization campaign was introduced in the remaining districts Western area urban and rural due to the ***ineffectiveness*** of the CDTI.”

*First, I am wondering what you mean by infectiveness of CDTI. I guess you are referring to challenges related to the implementation of CDTI in urban areas compared to rural areas. Secondly, I think this statement does not match with the reference provided which refers to high coverage of MDA of lymphatic filariasis which is in contradiction with the effectiveness of CDTI. Could you please look at this?*

**Methods**

3. On this section, I think there is a need having a brief introduction for this section. Could you please present in one or two sentences the methods used in your paper?
4. I think the first two headings of your methods section “**mass drug administration**” and “**epidemiological and programmatic coverage assessments**” could be brought back to the introduction. In the revised introduction section, you can briefly mention what was done in terms of Mass drug administration and treatment coverage. Since you clearly mentioned that epidemiological and programmatic surveys were published previously, this support also the suggestion of mentioning what was done in terms of epidemiological and programmatic coverage assessments in the introduction section.
5. You used in these section, the following acronyms/abbreviations **CHW**s”; “**re-pre-TAS**”, “NTDP “, “MOHS “, “**s**” . “**hr**”? What does each of them stand for? Please kindly refer to my comment on the use of acronyms and abbreviations (comment #1) .

**RESULTS**

**TAS in eight districts**

6. Could you please add the year of the survey in the title of table #1?

***Results of transmission assessment surveys in eight districts in four EU in 2017***

7. You mentioned that the number of FTS positive was well below the critical cut-off value for each evaluation unit as shown in Figure #2. I suggest that you add Table 1 as well
8. You mentioned that 49.3% and 50.7% of children examined during TAS were males and females respectively. No reader can see that in your table. Could you please split the column on “number of persons tested per EU” in three sub columns, one for males, one for females and the third one on total. This shows to show approximately the number of males and females to readers the above-mentioned statistics with regards to gender? (see suggestion below)

| **EU** | **District** | **Baseline mapping in 2005**  **(Prevalence (%) by ICT** | **Number of person tested per EU** | | | **Number of FTS positives** | | | **Critical cut-off value** |
| --- | --- | --- | --- | --- | --- | --- | --- | --- | --- |
|  |  |  | Males | Females | Total | Males | Females | Total |  |
| EU 1 | Kambia |  |  |  |  |  |  |  |  |
|  | Port Loko |  |  |  |  |  |  |  |  |
| EU2 | Bo |  |  |  |  |  |  |  |  |
|  | Pujehum |  |  |  |  |  |  |  |  |
| EU3 | Kono |  |  |  |  |  |  |  |  |
|  | Tonkolili |  |  |  |  |  |  |  |  |
| EU4 | Moyamba |  |  |  |  |  |  |  |  |
|  | Bonthe |  |  |  |  |  |  |  |  |
| Total | - | - | ?? | ?? | 7152 |  |  |  |  |

***Re-pre-TAS in four districts and pre-TAS in two districts***

9. The total of 3,994 persons is for districts targeted for Pre-TAS (4) and Re-pre-TAS (2) not for four districts during re-pre-TAS as highlighted in the paper. I suggest that you split the number in two and provide the number of people targeted for Re-Pre-TAS in four EUs and number of people targeted for Pre-TAS in two EUs.
10. Could you please refer to my comment# 8 to review table #2?
11. You presented the results of Pre-TAS and referred to table #3. Where is table #3? Were you referring to figure #3? If yes please kindly correct table #3 to figure #3.
12. Regarding Figure #3, one spot check village with FTS prevalence of 2.9 is missing in the figure. Could you please kindly add it?
13. With regards to pre-TAS results, you mentioned the individual antigenemia prevalence was 0.7% and 0.7% in West Area Urban qualifying for conducting TAS and 1.7% and 3.7% in Western Area rural failing to qualify for TAS as shown in **Figure #3**. I suggest you mention the table number as well after splitting your table#2 in two as I suggested above.
14. To differentiate Pre-TAS and re-pre-TAS in figure #3, could you please different colors?

***Microfilaria detection in the FTS positive cases***

15. The number of positive FTS in the four districts targeted for Re-pre-TAS is 278 not 296. Could you please check and confirm the provided number (296) in the paper?

**DISCUSSION**

16. You mentioned that only four districts failed the Pre-TAS. How about Western area rural?
17. You mentioned in your discussion section, key factors which could explain the ongoing transmission in Kailahum, Kenema, Koinadugu, Bombali and Western rural areas, which are high baseline prevalence in the border districts, traditional beliefs, treatment coverage issues due to population movement within the country/across borders, inaccurate denominators, internal migration, movement of pastoralists from the fullah ethnic groups, difficulty to keep to the appropriate treatment time, demotivation of CDDs, fear of side effects by communities. Do you have an idea of vectors species involved in the transmission of the infection? The knowledge of vectors responsible of the transmission is extremely important since not all of them have the same competence. Which species do you have in settings that passed TAS and which ones do you have in those that failed Pre-TAS and Re-pre- TAS?
18. The World Health Organization recommends integrated vectors control measures in the fight against lymphatic filariasis as alternative measures to combat Lymphatic filariasis. These include IRS and use of Long lasting insecticide treated bed nets (LLITNs). Do you have an idea of the use of IRS and LLITNs in these districts which passed TAS and in those where the transmission is still ongoing?

**CONCLUSION**

19. What are the limination of your study?
